# Supplementary material for: Context-enriched interactome powered by proteomics helps the identification of novel regulators of macrophage activation
Source: eLife. 2018 Oct 10;7:e37059. doi: 10.7554/eLife.37059 (PMC6179386; doi:10.7554/eLife.37059)
Supplement: Supplementary file 1. [file elife-37059-supp1.docx]

|  | Nodes/  Edges | Nodes/  Edges  (LCC) | Average  degree | Average  clustering  coefficient | Average  degree  (LCC) | Average  clustering  coefficient  (LCC) | Diameter  (LCC) | Average shortest  path length  (LCC) | Edge  density |
| --- | --- | --- | --- | --- | --- | --- | --- | --- | --- |
| **PPI** | 14213/170303 | 14115/170253 | 23.96 | 0.21 | 24.12 | 0.21 | 12 | 3.54 | 0.54% |
| **M0** | 2137/12448 | 2105/12424 | 11.65 | 0.29 | 11.80 | 0.29 | 21 | 8.13 | 1.06% |
| **M1** | 2118/23709 | 2074/23680 | 22.39 | 0.30 | 22.84 | 0.31 | 24 | 8.51 | 0.58% |
| **M2** | 2127/13024 | 2078/12994 | 12.25 | 0.28 | 12.50 | 0.29 | 31 | 8.67 | 0.17% |
