# Supplementary material for: Context-enriched interactome powered by proteomics helps the identification of novel regulators of macrophage activation
Source: eLife. 2018 Oct 10;7:e37059. doi: 10.7554/eLife.37059 (PMC6179386; doi:10.7554/eLife.37059)
Supplement: Supplementary file 2. [file elife-37059-supp2.docx]

|  | Bioplex v2.0  (OR, p-val) | Hein et al.  Cell 2015  (OR, p-val) | HI-II-14  (OR, p-val) | HI-III-16  (OR, p-val) |
| --- | --- | --- | --- | --- |
| **M(-)** | 1.46, 0.09 | 1.40, 0.06 | 2.71, 0.10 | 1.37, 0.42 |
| **M(IFNg)** | 0.93, 0.85 | 0.97, 0.94 | 0.96, 1.00 | 0.98, 1.00 |
| **M2(IL4)** | 2.07, 1.44E-04 | 1.71, 2.15E-03 | 1.93, 0.28 | 1.40, 0.42 |
