# Supplementary material for: Context-enriched interactome powered by proteomics helps the identification of novel regulators of macrophage activation
Source: eLife. 2018 Oct 10;7:e37059. doi: 10.7554/eLife.37059 (PMC6179386; doi:10.7554/eLife.37059)
Supplement: Supplementary file 3. [file elife-37059-supp3.docx]

| Top-ranked genes (p<0.01) | Inflammatome  (Overlap, OR, p-value) | InnateDB  (Overlap, OR, p-value) | CADGene  (Overlap, OR, p-value) |
| --- | --- | --- | --- |
| **PPI+M(-)** | 38, 3.67, 6.77e-10 | 39, 9.37, 2.52e-22 | 18, 6.17, 6.64e-09 |
| **PPI+M(IFNg)** | 32, 2.73, 4.30e-06 | 34, 7.26, 7.73e-17 | 15, 4.70, 2.61e-06 |
| **PPI+M2(IL4)** | 36, 3.23, 3.00e-08 | 32, 6.82, 2.70e-15 | 15, 4.77, 2.24e-06 |
